# Supplementary material for: Are carbon emissions trading and green financial instruments synergistic? -Comprehensive quantitative research based on content analysis
Source: PLoS One. 2024 Mar 7;19(3):e0298601. doi: 10.1371/journal.pone.0298601 (PMC10919601; doi:10.1371/journal.pone.0298601)
Supplement: S1 Data — (ZIP) [file pone.0298601.s001.zip › Data/地级市Z_F工作报告.zip!/░▓╗╒/┘±╓▌╩╨/└┤╫╘╣1⁄2╓┌║┼í╛┬φ┐╦╩2╛▌═°í┐_2013.docx]

各位代表：
　　现在，我代表市人民政府，向大会作政府工作报告，请予审议，并请市政协委员和其他列席人员提出意见。
　　2012年工作回顾
　　刚刚过去的2012年，在市委的坚强领导下，在市人大、市政协的监督支持下，我们紧紧依靠全市人民，深入贯彻落实科学发展观，坚持"稳中求进、好中求快、能快则快"的工作总基调，解放思想，攻坚克难，全力保持经济社会又好又快发展，圆满完成了市三届人大二次会议确定的目标任务。
　　预计，全市生产总值700亿元，同比增长11.5%；全部财政收入85.1亿元、增长36.8%，其中地方财政收入47.7亿元、增长40.3％；固定资产投资430亿元，增长30％；社会消费品零售总额303亿元，增长16％；进出口总额4.8亿美元，增长46％；实际利用外商直接投资3.5亿美元，增长40%；城镇居民人均可支配收入20630元，增长14%；农民人均纯收入6600元，增长17%；城镇新增就业6.66万人，城镇登记失业率4%以内；人口自然增长率8.47‰；单位生产总值能耗下降2.6%，主要污染物减排完成省下达任务。
　　过去一年，我们克服了经济下行压力加大等不利因素影响，全市经济逆势上扬，后发优势开始显现，主要指标增幅位居全省前列。特别值得一提的是：全部财政收入增幅位居全省第1位，总量跨上80亿元新台阶；粮食生产实现"九连丰"，成为全省首个"小麦亩产千斤市"；新增规模工业企业150家，古井集团实现销售收入超50亿元、上缴税金超15亿元的新跨越；百万人口区域中心城市、50万人口三县县城框架基本形成，市南部新区实现了三年初见成效的目标；成功举办了亳州市第一届体育运动会、2012年国际（亳州）中医药博览会暨第28届全国（亳州）中药材交易会。
　　一年来，主要做了以下工作：
　　（一）抓项目、稳增长，工业经济凸显新亮点。着力实施"125工业振兴计划"，全年在建工业项目470个，完成投资200亿元。预计规模工业实现增加值170亿元，增长18.3%；医药制造业实现产值115亿元；农产品加工业实现产值350亿元，煤炭开采及洗选业实现产值60亿元。投资超100亿元的利辛板集煤电一体化项目上马建设，投资30亿元的古井生态产业园投料生产，投资13.6亿元的江淮安驰15万辆微型汽车项目今年上半年将整车下线，投资10亿元的杉杉集团服饰加工项目基本建成。
　　（二）抓基础、建平台，城市面貌呈现新气象。着力推动工业化、城镇化融合发展，在中心城区实施城建项目82项，总投资89亿元，完成基础设施建设投资30亿元，10项重点工程完成年度任务。市南部新区铁道以西片区45平方公里骨干路网全部建成，现代中药产业创业基地A区投入使用，总投资40亿元的北部新城基础设施建设项目正式启动。亳芜现代产业园一期6平方公里范围内的8条道路建成通车，总投资56亿元的10个工业项目开工建设。各县区开发区各新扩区3平方公里，建成区总面积达70平方公里。房地产市场运行平稳，全年完成房地产开发投资85亿元。
　　（三）抓转型、优结构，"三农"工作迈出新步伐。大力实施农业农村"四百工程"和农民收入赶超计划。粮食生产再上新台阶，全年总产达88.5亿斤，我市荣获"全国粮食生产先进市"称号，三县一区全部荣获"全国粮食生产先进县（区）"称号。农业产业化转型升级步伐加快，龙头企业增加到2100个，农民专业合作社发展到3500家，土地流转面积达133万亩。农业结构不断优化，蔬菜播种面积达186万亩，中药材种植面积达110万亩，规模养殖场（大户）发展到18426家。农田水利建设成效明显，新增旱涝保收田18.65万亩。农业机械化水平稳步提高，农作物耕种收综合机械化率达83%。"千万亩森林增长工程"全面启动，完成人工造林7.36万亩，建设绿色长廊689公里，新完善农田林网91.8万亩。美好乡村建设扎实推进，建设新村432个，省、市级新农村建设示范村发展到151个。
　　（四）抓商贸、活流通，第三产业取得新成效。大力发展商贸物流业，投资15亿元的康美（亳州）华佗国际中药城一期工程全面竣工，投资5亿元的天运物流园投入使用，全年净增限额以上商贸流通企业103家，完成农家店信息化改造项目270个，新建农家店158家，家电下乡累计销售金额48.9亿元。着力促进外向型经济发展，新增有进出口实绩的企业30家，全省首家中药材进出口检测中心建成使用，海关驻亳工作组、国检驻亳办事机构正式成立。文化旅游业加快发展，全年实现旅游业总收入65亿元。
　　（五）抓招商、促投资，发展后劲得到新增强。扎实开展"招商引资推进年"活动，实际利用市外内资600亿元、增长50%以上。新引进亿元以上项目139个，总投资797亿元，宏基汽车零部件产业园等一批投资10亿元以上项目落户亳州；新增中小企业3500家、个体工商户2.6万户。继续开展"项目建设推进年"活动，谋划储备项目1400个、总投资3500亿元；238个省"861"行动计划项目完成投资190亿元，302个市重点项目完成投资301亿元。投资18亿元的省道307一级公路亳州至涡阳段开工建设，改造县乡公路200公里、农村公路危桥150座，建设村道502公里，国道105涡河大桥主桥面今年5月份可以合龙，500千伏伯阳输变电一期工程竣工投运。
　　（六）抓改革、破难题，加快发展增添新动力。扎实推进社会事业改革，非时政类报刊出版单位改革任务全面完成，县级公立医院改革顺利实施，邮政体制改革、事业单位分类改革和中小学教师职称改革有序开展。不断深化金融改革，药都银行成功改制为农村商业银行，徽商银行亳州支行升格为分行，交通银行亳州分行挂牌营业，蒙城农村商业银行和蒙城、利辛2家村镇银行获批筹建。着力破解资金、用地难题，全年新增贷款100亿元，市建投公司成功发行企业债券15亿元，古井集团12亿元中期票据获准发行；全市实现土地出让收入73亿元，新增建设用地3万亩。
　　（七）抓民生、促和谐，社会事业开创新局面。全年投入资金58.2亿元，31项民生工程完成年度任务。新开工廉租房2980套、公共租赁房8818套、棚户区改造住房13890套。累计建设农民工创业园26个，转移农村富余劳动力18.92万人。5项社会保险超额完成年度任务，城镇医疗保险实现市级统筹。新建、改扩建农村敬老院28所，农村五保集中供养能力达51%。大力发展教育事业，学前教育三年行动计划稳步实施，城乡义务教育均衡发展，普通高考本科达线率达48.3%，亳州中药科技学校和蒙城职教中心被命名为"国家中等职业教育改革发展示范学校"，亳州师专顺利通过省高职高专人才培养工作评估。科技创新能力不断提高，高新技术企业发展到25家，新增省级以上名牌产品7个、著名商标18件，引进博士10人、硕士75人、副高职称以上专业人才86人，投资2500余万元的科技创业孵化基地投入运行。医疗服务水平明显提升，全市新农合参合率100%，累计受益837.97万人次。文化事业繁荣发展，205个农家书屋、36个公共电子阅览室建成使用。全民健身计划深入实施，体育竞技水平不断提高，我市荣获"中国五禽戏之乡"称号。人口计生工作扎实开展，低生育水平保持稳定。审计、监察、物价、统计、气象、地震、科协、档案、地方志、红十字会、广播电视、新闻出版、妇女儿童、民族宗教、外事侨务、对台事务、扶贫开发、国有资产管理、无线电管理和机关事务管理等工作取得新进步，国防动员和民兵预备役建设、双拥、人防等工作取得新成效。
　　（八）抓效能、办实事，政府作风实现新转变。深入推进"五级书记带头大走访"活动，坚持开门接访和包保责任制，一批信访积案得到妥善化解。加强"平安亳州"建设，健全应急管理体系，严厉打击各类违法犯罪活动。继续开展"安全生产年"活动，安全生产形势平稳。加大食品药品监管力度，市场秩序进一步规范，质量稳步提升。自觉接受人大、政协监督，全年办复人大代表议案、建议287件，政协委员提案353件，满意率达95%以上。"六五"普法工作深入开展，第四届社区居民委员会换届工作顺利完成。大力实施"四项安全"工程，深入开展"发展环境优化年"和民主评议百名科长、模拟仿真督查等活动，严肃查处了一批违法违纪案件。
　　各位代表，回首2012年埋头苦干、攻坚克难的奋进历程，我们难以忘记，在关键时刻和重大问题上，是市委的坚强领导、科学决策，为我们做好工作提供了组织保证；我们难以忘记，在重点项目和基础建设中，是群众的理解支持、无私奉献，为我们加快发展提供了动力源泉；我们难以忘记，在压力加大、任务繁重时，是各位代表和委员的监督帮助、建言献策，为我们战胜困难提供了智慧力量。在此，我代表市人民政府，向全市广大工人、农民、知识分子，向全体干部和离退休老同志，向各民主党派、工商联、各人民团体和社会各界人士，向驻亳部队、武警官兵和政法干警，向中央、省驻亳各单位，向所有关心、支持亳州发展的朋友们，表示衷心的感谢并致以崇高的敬意！
　　在肯定成绩的同时，我们也清醒地看到，政府工作仍存在许多不足，经济社会发展还面临不少困难和问题。主要是：工业经济缺少大企业、大项目引领，农村基础设施还不完善，文化旅游产业开发还不够，资金、土地、环境等要素制约需进一步破解，一些地方私搭乱建、脏乱差现象仍然存在，少数干部领导科学发展能力不强，极个别干部作风不实、效率不高、为政不廉、违法乱纪等。我们一定要正视这些问题，直面群众关切，以敢于担当的气魄，以切实有力的举措，努力在解决问题中开拓前进！
　　2013年主要任务
　　2013年，是全面贯彻落实党的十八大精神的开局之年，是实施"十二五"规划承前启后的关键一年。从政策环境看，今年国家继续坚持稳中求进的工作总基调，实施积极的财政政策和稳健的货币政策，支持包括亳州在内的中原经济区加快发展，省委、省政府全力支持皖北崛起，我们将迎来新一轮大开放大发展的难得机遇。从发展基础看，经过近年来的不懈努力，我们已经具备了跨越赶超、加速崛起的良好条件，进入了一个潜力得以彰显、优势竞相迸发、可以大有作为的黄金发展期。我们要认清形势，抢抓机遇，奋力加快全面建成小康社会进程，让全市人民生活得更加富裕、更加和谐、更加幸福。
　　按照省委提出到2020年经济总量争先进位、人均水平进入中等、居民收入赶上全国的目标，与我市"十二五"规划相衔接，今年政府工作总体要求是：以邓小平理论、"三个代表"重要思想和科学发展观为指导，全面贯彻落实党的十八大精神，以科学发展为主题，以加快转变经济发展方式为主线，以稳中求进、好中求快、能快则快为主调，以提高经济增长质量和效益为核心，坚持"发展为上、民生为先、稳定为重，少说多做、真抓实干"，大力促进"四化同步"，努力打造"三大城市"，加快建设"美好亳州"，为全面建成小康社会奠定坚实基础。
　　经济社会发展的主要预期目标是：全市生产总值增长12%左右，总量突破800亿元；财政收入增长18%以上，总量突破100亿元；固定资产投资增长25%；社会消费品零售总额增长15%；进出口总额增长20%以上；实际利用外商直接投资增长30%；城镇居民人均可支配收入增长14%以上，农民人均纯收入增长15%以上；城镇登记失业率控制在4.2%以内；人口自然增长率控制在8.5‰以内；确保完成省政府下达的各项节能减排任务。
　　围绕上述目标，重点做好以下八个方面的工作：
　　（一）坚持创新驱动战略，加快新型工业化进程。
　　把创新驱动作为提高工业化水平的战略支撑，推动信息化和工业化深度融合，大力实施"125工业振兴计划"，力争规模企业达到700家、规模工业增加值增长18%以上。
　　实施特色产业升级工程。全力打造千亿元现代中药首位产业，推进修正药业等一批重点项目建设，确保年内医药制造业实现产值140亿元。全力打造500亿元白酒及农产品加工业，全面建成古井生态产业园，加快推进古井配套产业园、雨润食品工业园等重点项目，力争古井集团实现销售收入70亿元，全市农产品加工产值达到400亿元。全力打造200亿元煤电煤化工产业，重点抓好板集2×100万千瓦坑口电厂等项目建设。全力打造百亿元汽车及零部件制造业，加快江淮安驰新厂区等项目建设。全力打造百亿元劳动密集型产业，推进纺织服装、电子产品组装等重点行业集聚发展。培育发展战略性新兴产业，加快实施秸秆发电、垃圾发电项目，促进生物医药、电子信息、节能环保、新能源等新兴产业发展。
　　实施科技创新引领工程。着力构建技术创新体系，组织申报省以上各类科技计划项目30项，新申报认定高新技术企业5家，新增生产力促进中心2家、科技企业孵化器2家，完成专利申请1800件；新增名牌产品4个、省著名商标20件；建立院士工作站、博士后创新工作站。
　　实施园区转型发展工程。加快园区转型升级，确保5个省级开发区全年新入园项目100个以上，市南部新区、亳芜现代产业园和县、区开发区各新开工建设亿元以上工业项目10个，其中10亿元项目1个、5亿元项目2个。继续抓好全市31个乡镇工业功能区建设，力争新增规模企业60家以上。
　　（二）加强基础设施建设，提升特色城镇化水平。
　　把推进城镇化作为跨越赶超的重要抓手，围绕建设百万人口区域中心城市目标，以城镇基础建设为重点，构建功能完善、布局优化的宜居宜业城市。
　　完善中心城区功能。抓好各类建设规划修编工作，加快建设市规划馆。今年计划实施主城区续建项目166个、总投资193.8亿元、年度计划投资121.4亿元，新开工项目94个、总投资259.4亿元、年度计划投资61.1亿元。突出抓好10项重点工程：（1）实施市南部新区主干路网完善工程。重点实施魏武大道、希夷大道南延工程，加快文采路、火神路、世纪大道建设，打通南扩一路。（2）实施市北部新城基础设施建设工程。重点抓好路网建设、污水处理和管线铺设等工程。（3）实施城区水系治理和公园建设工程。重点抓好亳城新河、凤尾沟、宋汤河等治理项目，加快龙凤公园、气象科普公园、郑店子中华药都养生园和涡河景观带二期工程建设。（4）实施老城区基础设施完善工程。开工建设涡河人民大桥新桥，完成西一环改造项目，加快涡河南岸和城中村拆迁开发进度，对部分干道进行白加黑改造，抓好部分小街巷和20座公厕改造工程。（5）启动建设120万平方米的4个安置还原小区。（6）实施五位一体公共活动中心建设工程。建设市职工、青少年、妇女儿童、老年活动中心和文化馆。（7）实施文化古迹保护工程。修缮保护曹操八角台遗址、道德中宫、薛阁塔及观音山汉墓和北关历史街区重点古迹。（8）启动建设绕城快速通道西线和南线工程。（9）实施市垃圾处理场和自来水厂新建工程。（10）启动市政务新区、市体育馆和市城建档案馆建设工程。
　　推进精品县城建设。三县县城要按照产城一体的思路，一手抓旧城改造，一手抓新区扩张，今年各新扩区3平方公里。重点支持涡阳县加快南部新城建设，打造现代新城集中展示区；支持蒙城县启动建设城南新区西片区，打造森林城市；支持利辛县加快建设滨河新区，打造生态滨水城市。
　　提升城市管理水平。实施数字城市建设工程，重点做好数字城管、数字社区、数字交通等工作。实施城市道路畅通工程，着力解决交通秩序混乱、车辆乱停乱放、违章事故频发等突出问题。实施市容环境整治工程，着力解决占道经营、私搭乱建、乱堆乱放等突出问题。实施城区美化亮化工程，着力解决建筑立面、沿街灯饰、景观绿化等方面存在的突出问题。
　　（三）加快发展现代农业，推动美好乡村建设。
　　把"三农"工作作为加速崛起的重中之重，加快推进农业产业化转型升级，大力实施农民收入赶超计划，促进农业增效、农民增收。
　　调整优化农业结构。稳步提升粮食生产能力，力争全年实现粮食总产90亿斤；开展创建全省首个"吨粮市"活动，力争3年内实现目标。加快实施"畜牧业升级计划"，力争规模养殖比重达到75%。加快建设涡河中药材种植产业带，中药材种植面积稳定在100万亩以上。加快发展特色蔬菜和设施蔬菜，力争蔬菜种植面积达190万亩，新增设施蔬菜8万亩。新增无公害农产品产地、绿色食品原料基地8个，新认证农业"三品"20个。启动建设市农产品质量检测中心。
　　推进农业产业化转型升级。以涡阳国家级现代农业示范区为重点，突出抓好10个现代农业核心区建设。继续实施"百家龙头企业提升工程"，力争龙头企业销售收入达240亿元。深入开展农业产业化示范区创建活动，力争市级以上产业化示范区产值达到200亿元以上。扎实推进农民专业合作社示范社建设，力争各类农民专业合作社总数达到4000家。大力实施农业机械化推进工程，力争农作物耕种收综合机械化率达到85%以上。
　　加快农田水利建设。开展农田水利建设高潮年活动，加大中小河流治理和病险涵闸除险加固工程推进力度，完成芡河、油河等河流和灌区治理任务；完善水利设施管护机制，建设高标准农田水利受益区10.93万亩；做好引淮济亳、洼地治理、淮干堤防、大型泵站等项目前期工作。推进农村饮水安全工程建设，力争全市农村饮水安全覆盖率达到60%。
　　全面建设美好乡村。高起点编制美好乡村建设规划，发挥财政资金引导作用，重点打造涡河美好乡村百里长廊和西淝河美好乡村示范带，抓好121个中心村建设，治理改造620个自然村。创新农业生产经营机制，推进农村土地向规模经营主体集中，累计完成土地流转面积160万亩。不断加强农村基础设施建设，加固改造农村公路危桥150座、改造县乡公路200公里。大力发展农村公共事业，深入开展"文明村镇创建"活动，完成12个乡镇农村清洁工程。
　　（四）加快发展现代服务业，促进第三产业提质增效。
　　坚持生产性服务业与生活性服务业并重、现代服务业与传统服务业并举，促进服务业发展提速、比重提高、水平提升，第三产业增加值增长11%以上。
　　大力发展商贸物流业。深入实施"万村千乡市场工程"和"新网工程"，加快改造提升农贸市场，鼓励连锁经营企业向农村延伸。着力完善放心肉、放心菜、放心店、放心早餐、放心家政"五位一体"便民服务体系，大力推进国家级中药材流通追溯体系和家政服务体系试点建设。加快推进大唐国际城、广齐城市广场等重点项目建设，积极引进家乐福、大润发等知名企业进驻亳州。继续抓好康美（亳州）华佗国际中药城二期工程、天津医药亳州天泉医药物流配送等项目建设，培育发展第三方物流。
　　积极发展文化旅游业。抓好花戏楼、华祖庵和老子文化生态园等景点改造升级工程，发展以印象江南为代表的乡村旅游，引导创意设计、演艺娱乐、影视服务、数字动漫、文化会展等产业发展，创建3A级以上旅游景区2家、3星级以上农家乐3家，力争全市旅游总收入增长20%以上。
　　培育壮大新兴服务业。加快发展保洁、租赁、旧货市场和二手车流通等服务业，培育市场调研、设计咨询、信息服务等中介行业，规范保险、会计、典当、拍卖、公证等服务机构，促进会议会展、文化娱乐、体育健身、医疗保健等服务业发展。办好2013年国际（亳州）中医药博览会暨第29届全国（亳州）中药材交易会。
　　（五）发挥投资拉动效应，抓好招商引资和项目建设。
　　深入开展"招商引资攻坚年"、"项目建设攻坚年"活动，大力发展外向型经济，不断增强发展后劲。
　　扩大开放引进项目。进一步解放思想，依托优势资源、优惠政策和优良环境，充分发挥药、煤、粮、酒、市场、劳动力等优势，以工业招商为重点，以产业招商为主线，创新招商方式，实施精准招商，扩大与央企、省企和知名民企合作，确保全年招商引资实际到位资金增长30%以上，引进市外内资800亿元以上。大力促进外向型经济发展，加快现代中药产业外贸转型升级示范基地建设，扩大农副产品、服装鞋帽等产品出口，力争新增外贸进出口实绩企业30家。
　　加大力度建设项目。坚持大中小项目协调推进，实施亿元以上项目160个、10亿元以上项目40个。加快省道307线一级公路亳州至涡阳段建设进度，完成亳阜高速公路出入口改造建设任务。开工建设220千伏漆园输变电工程，确保220千伏赵桥输变电等项目竣工投产。争取开工建设济祁高速公路亳州段工程，做好省道202线、省道307线一级公路涡阳至蒙城段、国道105线一级公路以及涡河航道整治、商杭高铁、亳州机场项目前期工作。围绕国家宏观政策、产业导向和投资需求，完善充实项目储备库，力争全年新谋划项目不少于1000个、总投资3000亿元以上。
　　（六）深化重点领域改革，增强加快发展活力。
　　加快重点领域和关键环节改革，不断创新完善有利于推动科学发展、和谐发展、跨越发展的体制机制。
　　全面深化国企改革。以古井集团、市建投公司为重点，完善法人治理结构，建立市场化薪酬制度，加强经营业绩考核，强化国有资产监管，着力提高企业核心竞争力。全力支持市、县区交通投资有限公司加快发展。
　　不断加快财税、金融改革。加强公共财政体系建设，推进预算绩效管理；巩固扩大"营改增"试点成果；继续实施城镇居民收入倍增和农民收入赶超计划，千方百计增加城乡居民特别是中低收入者收入。不断完善地方金融体系，完成蒙城农村商业银行和徽商银行利辛支行成立工作，组建三县村镇银行，加快涡阳、利辛联社改制步伐，力争完成新增贷款120亿元，实现直接融资30亿元以上。
　　继续推进卫生、教育改革。进一步巩固基层医药卫生体制综合改革成果，全面完成县级公立医院改革评估验收工作。推进基础教育三项改革，促进县域义务教育均衡发展，完善农民工子女教育体制机制，规范办学行为，减轻学生课业负担。抓好中小学教师职称改革试点工作。
　　大力发展非公有制经济。毫不动摇鼓励、支持、引导非公有制经济加快发展，把非公有制经济发展纳入政府考核体系，扶持现有企业做大做强，进一步放宽创业条件，着力解决好企业融资、用地、用工等难题，全年新增中小企业2100户、个体工商户2万户。
　　（七）抓好生态文明建设，提高持续发展能力。
　　牢固树立用发展保护生态的观念，坚定不移地推进资源节约型和环境友好型社会建设。
　　切实抓好节能减排。认真落实国家能源消费强度和总量双控制要求，坚决淘汰落后产能，突出抓好重点企业的节能降耗工作。实行减排工作责任制，加强环保重点项目建设，确保涡北等5家污水处理厂按时建成并实现减排。倡导"文明、节约、绿色、低碳"理念，促进生态环境持续改善。
　　推进土地集约利用。严守耕地保护红线，严格土地用途管制，强化"空心村"和采煤塌陷区治理，引导农民相对集中建房，促进工业项目节约集约用地。继续抓好城乡建设用地增减挂钩工作，加大基本农田整理力度，大力推进土地复垦开发，努力完成78万亩高标准基本农田任务，确保全年补充耕地6000亩。
　　加快生态亳州建设。实施千万亩森林增长工程，千方百计拓展造林空间，最大限度挖掘造林潜力，完成人工造林10.47万亩，建设绿色长廊600公里。全面推动"生态县"创建工作，启动城市绿道示范段建设。
　　（八）加强社会建设，保持社会和谐稳定。
　　围绕社会和谐稳定，切实解决事关群众切身利益的实际问题，努力提升人民群众幸福指数。
　　着力保障和改善民生。实施31项民生工程，确保完成年度目标任务。实施创业富民工程，争创省级创业型城市，突出做好高校毕业生、农村转移劳动力、城镇困难人员、退役军人等重点人群的就业工作，新增城镇就业岗位3万个，转移农村富余劳动力7.2万人。加强社会保障体系建设，做好5项社会保险扩面征缴工作。完善社会救助体系，进一步提高城乡低保标准。推进社会养老服务体系建设，农村五保集中供养能力达到59%以上。继续加快保障性住房建设，全年新开工保障性住房3.7万套，续建2.5万套。保持物价水平基本稳定。继续抓好扶贫开发工作。
　　加快社会事业发展。全面完成学前教育三年行动计划，大力推进义务教育学校标准化建设，不断扩大优质高中教育资源，全力抓好职教园区和亳州幼师新校区建设，支持亳州师专加快升格步伐。健全城乡卫生服务体系，推进市人民医院新院二期工程和市精神病医院建设，力争新农合参合率稳定在95%以上；实施中医药服务能力提升工程，大力发展中医药事业。深入推进文化惠民工程，加快市图书馆建设，提高乡镇综合文化站、农家书屋等公共文化资源使用效益。加强重点文物古迹保护，抓好非物质文化遗产传承工作。坚持计划生育基本国策，提高出生人口素质。推动群众体育和竞技体育全面发展，力争实现五禽戏百万人习练目标。大力发展社会福利、残疾人和慈善事业，保障妇女儿童权益，启动建设市、县（区）残疾人综合服务中心。完善防灾减灾体系，提高气象灾害防御能力。做好第三次全国经济普查工作。加强外事、侨务、对台、防震减灾、无线电管理和机关事务管理工作，推动档案、地方志、红十字会、老龄事业健康发展。
　　加强和创新社会管理。坚持社会管理重心下移，强化社区自治服务功能，全面实行居住证制度，稳妥推进户籍管理改革。强化宗教事务管理，促进民族和谐发展。继续巩固"五级书记带头大走访"活动成果，建成市群众来访接待中心，健全联系群众长效机制，加大矛盾纠纷排查化解力度，着力化解信访积案。深入推进公民道德建设工程，加强政务诚信、商务诚信和司法公信建设，培育全社会知荣辱、讲正气、作奉献、促和谐的良好风尚。
　　完善公共安全体系。扎实开展"六五"普法，提高全民法制意识。深入开展"安全生产年"活动，坚决杜绝重特大事故发生。强化食品药品安全监管，建设国家中药材质量监督检验中心，建立质量可查、可控、可追溯体系。健全应急管理体系，做好消防安全工作，提高预防和处置突发事件能力。继续推进"平安亳州"建设，完善社会治安防控体系，依法预防和打击各类违法犯罪活动。不断加强国防教育、国防动员、双拥优抚和人民防空工作，推进国防后备力量建设。
　　全面加强政府自身建设
　　在新的一年里，我们要按照中央、省委和市委关于改进工作作风、密切联系群众的要求，以敢为、能为、有为的精神，强化执行力，提升公信力，更好地为全市人民服务、对全市人民负责。
　　（一）牢记奋斗目标。人民对美好生活的向往就是我们的奋斗目标。任何时候都要把人民利益放在第一位，始终与人民心连心、同呼吸、共命运，永远站在群众立场上思考问题，永远帮助群众排忧解难，永远做人民群众的公仆，努力创造亳州人民的美好幸福生活。要牢记为民宗旨，了解社情民意，倾听群众呼声。要致力改善民生，把政府工作的重点、政策支持的重点、财力保障的重点，向社会事业、民生工程等方面倾斜，切实解决群众关心的征地拆迁、住房保障、看病就医、子女入学等突出问题。
　　（二）切实改进作风。全市政府系统要切实改进工作作风，深入调查研究，精简会议文件，解决实际问题。要坚持依法行政，推进法治政府建设，严格按法定程序、法定权限行使职权。要深化政务公开，完善重大事项公众参与、专家论证、风险评估、合法性审查和政府集体决策相结合的制度，提高决策科学化、民主化水平。要自觉接受人大、政协的监督，认真听取各民主党派、工商联、无党派人士的意见，主动接受公众和新闻媒体监督，支持工会、共青团、妇联充分发挥桥梁纽带作用。要继续开展"发展环境优化年"和民主评议百名科长、模拟仿真督查活动，着力整治庸懒散奢等不良风气，不断提高政府工作执行力。
　　（三）奋力真抓实干。"空谈误国，实干兴邦"。我们要敢于担当，以舍我其谁的精神，勇于挑重担，敢于负责任，想干事、不怕事、干成事、不出事。要攻坚克难，面对各种矛盾不回避、不退缩，面对急难险重任务不推诿、不扯皮，做到迎难而上，敢于碰硬、善于决断，创造性地开展工作。要苦干实干，全面推行"一线工作法"，从一桩桩、一件件实事抓起，各司其职，各负其责，少说多做，埋头工作，以实实在在的业绩赢得群众的拥护和支持。
　　（四）必须廉洁从政。清正廉洁是政府工作人员的从政底线。要全面推进惩治和预防腐败体系建设，严格落实党风廉政建设责任制，完善领导干部制约监督机制，加强廉政风险防控工作，落实行政问责制度，切实做到干部清正、政府清廉、政治清明。要厉行勤俭节约，严格控制"三公"支出，把有限的财力用在促发展、保重点、惠民生上。要加强审计监督和行政监察，深入开展重点领域专项整治，坚决纠正行业不正之风，严厉查办违法违纪案件，确保生产安全、工程安全、资金安全和干部安全。
　　各位代表，站在新的起点，我们信心倍增；展望美好未来，我们豪情满怀！让我们更加紧密地团结在以习近平同志为总书记的党中央周围，在省委、省政府和市委的坚强领导下，紧紧依靠全市人民，大思路谋划发展，大手笔推进建设，大成效实现跨越，万众一心，开拓进取，为加快建设美好亳州、全面建成小康社会而努力奋斗！
　　《政府工作报告》名词术语解释
　　1."125工业振兴计划"--到2015年，全市培育规模工业企业1000家以上，打造现代中药、白酒及农产品加工等2个500亿元产业集群，建设5个百亿元产业园区。
　　2.农业农村"四百工程"--到2015年，全市粮食总产稳定在100亿斤以上，中药材种植面积发展到100万亩以上，亿元以上农业产业化龙头企业发展到100家，转移农村劳动力100万人以上。
　　3.农民收入赶超计划--到2015年，全市农民人均纯收入突破10000元，赶上或超过全省平均水平。
　　4.千万亩森林增长工程--《安徽省千万亩森林增长工程总体规划（2012-2016年）》提出，到2016年，新增森林面积1000万亩，全省森林覆盖率达到33%。
　　5.美好乡村--根据《中共安徽省委安徽省人民政府关于全面推进美好乡村建设的决定》，我市已全面启动美好乡村建设，努力打造"生态宜居村庄美、兴业富民生活美、文明和谐乡风美"的农民幸福家园，从2013年开始，每年重点培育建设121个中心村，治理改造620个自然村，到2016年，力争全市40%以上的中心村达到美好乡村建设要求；到2020年，力争全市80%以上的中心村达到美好乡村建设要求。
　　6.省"861"行动计划--省政府提出，从2012年起调整实施"861"行动计划，重点打造电子信息、汽车和装备制造、材料和新材料、食品医药、纺织服装、能源和新能源、文化产业、现代服务业等八大产业，着力推进综合水利、交通网络、生态环保、城镇化、人才和民生工程等六大基础工程，努力实现未来五年地区生产总值翻一番的目标。
　　7."四化同步"--党的十八大报告指出，坚持走中国特色新型工业化、信息化、城镇化、农业现代化道路，推动信息化和工业化深度融合、工业化和城镇化良性互动、城镇化和农业现代化相互协调，促进工业化、信息化、城镇化、农业现代化同步发展。
　　8."三大城市"--市委提出，努力把亳州建设成为百万人口的区域中心城市、特色鲜明的新型工业城市、宜居宜业的和谐幸福城市。
　　9."吨粮市"--市委、市政府研究决定，从2013年起，在成功创建小麦亩产千斤市的基础上，继续实施粮食增产计划，力争到2015年，全市粮食平均亩产达1000公斤，创建全省首个"吨粮市"。
　　10."畜牧业升级计划"--以建设现代畜牧业为目标，着力推进标准化规模养殖、良种繁育、畜产品质量安全、产业化经营和动物疫病防控水平等全面升级，到2015年，肉蛋奶总产达到50万吨，规模养殖比重达到80％，畜牧业产值100亿元。
　　11.农业"三品"--农业部组织实施的我国农产品质量安全认证的基本类型，属于安全农产品的范畴，指无公害农产品、绿色食品和有机食品。
　　12.城镇居民收入倍增计划--到2015年，力争全市城镇居民人均可支配收入在2010年基础上翻一番。
　　13.基础教育三项改革--推进县域义务教育均衡发展改革、完善农民工子女教育体制机制改革、规范办学行为减轻学生课业负担改革。
　　14.城市绿道--省政府制定了关于实施绿道建设的意见，提出2013年6月底前，各市、县要编制完成绿道总体规划，启动城市绿道示范段建设。到2016年，全省设区城市和县（市）城区建成绿道3000公里以上，全省城市绿道框架基本形成。
